# Antimicrobial Effect of *Zophobas morio* Hemolymph against Bovine Mastitis Pathogens

**DOI:** 10.3390/microorganisms8101488

**Published:** 2020-09-28

**Authors:** Mengze Du, Xiaodan Liu, Jiajia Xu, Shuxian Li, Shenghua Wang, Yaohong Zhu, Jiufeng Wang

**Affiliations:** Department of Veterinary Clinical Sciences, College of Veterinary Medicine, China Agricultural University, Beijing 100193, China; dumengze@126.com (M.D.); lxd20200228@163.com (X.L.); jajaxu@126.com (J.X.); 18813011821@163.com (S.L.); wsh20200817@163.com (S.W.); zhu_yaohong@hotmail.com (Y.Z.)

**Keywords:** *Zophobas morio* 1, antimicrobial hemolymph 2, bovine mastitis 3, inflammasome 4, pyroptosis 5

## Abstract

Coliforms and *Staphylococcus* spp. infections are the leading causes of bovine mastitis. Despite extensive research and development in antibiotics, they have remained inadequately effective in treating bovine mastitis induced by multiple pathogen infection. In the present study, we showed the protective effect of *Zophobas morio* (*Z. morio*) hemolymph on bovine mammary epithelial cells against bacterial infection. *Z. morio* hemolymph directly kills both Gram-positive and Gram-negative bacteria through membrane permeation and prevents the adhesion of *E. coli* or the clinically isolated *S. simulans* strain to bovine mammary epithelial (MAC-T) cells. In addition, *Z. morio* hemolymph downregulates the expression of nucleotide-binding oligomerization domain (NOD)-like receptor family member pyrin domain-containing protein 3 (NLRP3), caspase-1, and NLRP6, as well as inhibits the secretion of interleukin-1β (IL-1β) and IL-18, which attenuates *E. coli* or *S. simulans*-induced pyroptosis. Overall, our results suggest the potential role of *Z. morio* hemolymph as a novel therapeutic candidate for bovine mastitis.

## 1. Introduction

Bovine mastitis (BM) is the most prevalent disease affecting the dairy industry worldwide, and it largely impacts both the productivity and quality of dairy products, which lead to huge economical loss [1]. *Staphylococcus* spp. strains are considered the most common pathogen for BM [2]. In a survey conducted from 2015 to 2017, the isolation rate of *Streptococcus* spp. in 6305 samples from Shandong, China, was as high as 57.9% [3]. In addition to that, *E. coli* is also involved in BM induction, where an epidemiological study showed that *E. coli* is present in 14.4% of affected dairy cows [4]. In recent years, antibiotics have been used as the major treatment for BM, but overuse of antibiotics may leave residues in dairy products, which promote antibiotic resistance [5]. As a widely used antibiotic drug, cefotaxime was shown to have little improvement in cure rate, bacterial clearance, or bacterial load in Gram-negative bacteria-induced BM [6]. While others have reported that in Gram-positive bacterial infection-induced BM, antibiotic treatment decreases somatic cell count (scc) in milk products, the overall quality is still affected [7]. In addition, after mastitis treatment with gentamicin, which is a very effective antibiotic drug, it leads to the emergence of multidrug-resistant strains, as well as increasing the risk of the presence of this drug in the milk [8]. Regarding this, it is critical to develop a new class of drugs that are effective in treating both Gram-positive and Gram-negative bacteria-induced BM.

Inflammasomes are supramolecular cellular complexes driving multiple innate immune responses, which include inflammatory caspases activation, proinflammatory interleukin 1 beta (IL-1β) and IL-18 maturation, as well as pyroptosis [9]. Recent studies have shown that transfected bacterial endotoxin LPS triggers noncanonical inflammasome activation [10]. NLRP6, a nucleotide-binding oligomerization domain (NOD)-like receptor family member, can also form inflammasome upon recognition of intestinal microbes [11]. Emerging evidence of interactions between inflammasomes and bacterial ligands emphasized the importance of inflammasome activation in regulating host immune response against bacterial infection; however, the specific mechanism of inflammasome activation in BM remains to be elucidated.

Inflammasome activation is generally considered beneficial for bacterial clearance; however, exacerbating activation of inflammasomes may cause serious side effects [12]. Previous studies have shown that inflammation and cell damage are critical in BM pathogenesis [13], but attenuation of ASC-independent NLRP3 inflammasome activation, on the other hand, significantly ameliorates BM severity [14]. Therefore, targeting inflammasome activity should be a potential solution in treating BM.

In recent years, many strategies have been developed to treat BM by enhancing the host immune response. Specifically, overexpression of antimicrobial peptides in the mammary gland was shown to be effective, which indicated the potential of developing antimicrobial peptide drugs in treating BM [15]. With the lack of adaptive immunity, insects have developed two efficient innate immune systems in defending themselves against attack outside: Phagocytosis and encapsulation by specialized hemocytes; direct killing by enzymes and antimicrobial peptides secreted in hemolymph [16]. Earlier studies found that injection of bacteria into pupae of cecropia moth induces the secretion of antimicrobial molecules in hemolymph [17]. It was followed by successful extraction of two kinds of antimicrobial peptides from *Zophobas atratus* (*Z. atratus*), and their activity in killing both Gram-positive and Gram-negative bacteria was demonstrated as well [18].

In the present study, we performed a similar bacterial injecting strategy to induce antimicrobial compounds in the hemolymph of *Z. morio*, a widely extant species in China that belongs to the same genus with *Z. atratu*. The safety and antibacterial activity of hemolymph were assessed through a series of in vitro models. We found that *Z. morio* hemolymph is effective in both the direct killing of BM-inducing pathogens and downregulation of inflammasome genes expression and/or activity, which leads to the attenuation of BM severity. Overall, our study highlighted the potential of *Z. morio* hemolymph as a novel drug candidate in treating BM.

## 2. Materials and Methods

### 2.1. Bacterial Strains and Growth Conditions

*Escherichia coli* (*E. coli*, CVCC1450), *E. coli* (ATCC25922), *Staphylococcus aureus* (*S. aureus*, ATCC25923), *Proteus vulgaris* (*P. vulgaris*, CVCC1971), *Clostridium perfringens* (*C. perfringens*, CVCC1125), *Streptococcus suis* (*S. suis*, CVCC3307), and *Salmonella typhimurium* (*S. typhimurium*, ATCC14028) were purchased from the China Institute of Veterinary Drug Center (Beijing, China) and cultured in the lab with luria-bertani (LB) broth (Oxoid, Basingstoke, England). *Klebsiella pneumoniae* (*K. pneumoniae*) and *Staphylococcus simulans* (*S. simulans* No.11582) were isolated from milk samples of dairy cows with subclinical mastitis from a local farm and cultured in the lab with caton-adjusted mueller-hinton agar (CAMH-A) or caton-adjusted mueller-hinton broth (CAMH-B) (Oxoid, Basingstoke, England).

### 2.2. Z. morio Immunization and Hemolymph Collection

As previously described [18], 3rd instar larvae of *Z. morio* were injected with 1 μL of heat-killed, overnight culture of *E. coli* or other bacterial strains (approximately 1 × 10^7^ cells per injection). Then, 24 h later, injected insects were chilled for 1 min in ice-cold water, and approximately 30 μL of hemolymph was recovered by sectioning the metathoracic leg and gently squeezing the abdomen cavity. Harvested hemolymph was pooled in precooled plastic tubes and boiled for 10 min. After centrifugation at 20,000× *g* for 30 min at 4 °C, the cell-free hemolymph was collected and filled through a 0.2 μm filter. Hemolymph from larvae without infection was also collected as a negative control. For quality test, 10 mg of hemolymph was run in 10% SDS-PAGE and analyzed.

After qualification, cell-free hemolymph was frozen at −80 °C for 1 h and then sublimated for 6 h under a pressure of 0.5 mbar and trap temperature of −40 °C. Lyophilized hemolymph was stored at −20 °C for long-term storage and re-dissolved in an appropriate volume of sterile water for the inhibition zone tests and biostability determination.

For cell assays, qualified, cell-free hemolymph was centrifugated (5000× *g*, 15 min) with an Amicon Ultra-100 centrifugal filter (Millipore, MA, USA). The filtrated hemolymph was centrifugated (5000× *g*, 15 min) by an Amicon Ultra-30 centrifugal filter (Millipore, MA, USA), and the supernatant hemolymph was used for the cell experiment. For long-term storage, supernatant was lyophilized as described above.

### 2.3. Minimum Inhibitory Concentration (MIC) and Minimum Bactericidal Concentration (MBC) Evaluations

A bacterial susceptibility assay was performed to primarily compare the susceptibility of various bacterial strains against *Z. morio* hemolymph. As previously described [18], 6 mm-diameter wells were cut out in sterile petri dishes with 7.5 mL of CAMH-A medium, and 20 μL of hemolymph was added in each well. Later, log-phase bacteria were evenly coated on the plate (approximately 1 × 10^6^ cells/plate). Plates were incubated overnight at 37 °C, and the diameters of the clear zones were recorded. *E. coli* ATCC25922 served as a reference control. Three experiments were carried out in duplicate.

For MIC determination, each bacteria strain (1 × 10^8^ CFU/mL or OD_600_ of 0.5) was diluted 1:1000 in CAMH-B medium and added to a 96-well plate (100 μL/well). Then, 100 μL of serially diluted hemolymph (ultra-filtrated and lyophilized) was added. The MIC value was recorded after 18 h of incubation. *E. coli* ATCC25922 was used as a reference control. Three experiments were carried out in duplicate.

The MBC of *Z. morio* hemolymph was determined after MIC. A 10 μL sample from each well of the MIC plates was collected and transferred into a fresh CAMH-A plate. Plates were incubated at 37 °C for 18 to 24 h, and the MBC value was recorded. *E. coli* ATCC25922 was used as the reference control. Three experiments were carried out in duplicate.

### 2.4. Biostability Determination of Z. morio Hemolymph

#### 2.4.1. Effect of High Temperature on the Bioactivity of *Z. morio* Hemolymph

To assess the effect of high temperature on the antibacterial activity of *Z. morio* hemolymph, hemolymph samples were boiled in a water bath for 5 to 30 min. In addition, 20 μL of boiled hemolymph was added into the CAMH-A plate with *E. coli* (ATCC25922) and incubated overnight at 37 °C. The diameter of clear zones was recorded. Three experiments were carried out in duplicate.

#### 2.4.2. Effect of Repeated Freeze–Thaw Cycle on the Bioactivity of *Z. morio* Hemolymph

Hemolymph samples were frozen and thawed 6 times. Then, 20 μL of treated hemolymph was added into the CAMH-A plate with *E. coli* (ATCC25922) and incubated overnight at 37 °C. The diameter of clear zones was recorded. Three experiments were carried out in duplicate.

#### 2.4.3. Effect of Dissolving in Cow mIlk on the Bioactivity of *Z. morio* Hemolymph

Lyophilized *Z. morio* hemolymph samples were dissolved in the same volume of milk where it was extracted from. Further, 20 μL of dissolved hemolymph was added into the CAMH-A plate with *E. coli* (ATCC25922) and incubated overnight at 37 °C. The diameter of clear zones was recorded. Three experiments were carried out in duplicate.

#### 2.4.4. Effect of UV Exposure on the Bioactivity of *Z. morio* Hemolymph

Here, 500 μL of hemolymph was added into 24-well cell culture plates and exposed under UV light for 5 to 40 min. Then, 20 μL of UV-treated hemolymph was added into the CAMH-A plate with *E. coli* (ATCC25922) and incubated overnight at 37 °C. The diameter of clear zones was recorded. Three experiments were carried out in duplicate.

### 2.5. Time Course Determination of Z. morio Hemolymph’s Inhibitory Activity

Overnight-cultured *E. coli* CVCC1450 or *S. aureus* ATCC25923 was diluted in CAMH-B media (1 × 10^5^ CFU/mL) with 1 MIC of *Z. morio* hemolymph (0.5 mg/mL for *E. coli* CVCC1450 and 1 mg/mL for *S. aureus* ATCC25923). In addition, 1 MIC of gentamicin (0.5 μg/mL) was used as a control drug in both assays. Bacteria were cultured at 37 °C with constant shaking and the OD_595_ was recorded at 1, 2, 3, 4, 5, 6, 7, or 8 h after drug addition. Three experiments were carried out in duplicate.

### 2.6. Time Course Determination of Z. morio Hemolymph’s Bactericidal Activity

*E. coli* strains CVCC1450 and *S. aureus* ATCC25923 were used in this assay. After overnight culture, bacteria were diluted 1:1000 in 1 mL CAMH-B medium and cultured subsequently at 37 °C with constant shaking, and 10 MIC of *Z. morio* hemolymph (5 mg/mL for *E. coli* CVCC1450 and 10 mg/mL for *S. aureus* ATCC25923) was added 4 or 8 h later. Then, 10 MIC of gentamicin (5 μg/mL) and enrofloxacin (1.25 μg/mL) were used as control drugs in the *E. coli* group, and gentamicin (5 μg/mL), enrofloxacin (2.5 μg/mL), and Ampicillin (20 μg/mL) were used as control drugs in the *S. aureus* group. Next, 100 μL of bacteria culture was collected at 2, 4, 6, 8, 10, 12, 14, 16, 18, or 20 h after drug addition and evenly coated on CAMH-A plates after appropriate folds of dilution. Plates were incubated at 37 °C overnight and the number of colonies were counted. Three experiments were carried out in duplicate.

### 2.7. Scanning Electron Microscopy

As previously described [19], bacteria in the exponential phase were diluted in phosphate-buffered saline (PBS) to reach a final concentration of 10^8^ CFU/mL. Bacterial samples were then treated with 1 MBC of *Z. morio* hemolymph (1 mg/mL for *E. coli* CVCC1450 and 1 mg/mL for *S. aureus* ATCC25923) at 37 °C for 30 min. After that, bacterial samples were collected by centrifugation (5000× *g*, 10 min) and fixed according to the following procedure. Cells were fixed at 4 °C overnight with 3% glutaraldehyde in 1 M phosphate buffer (pH 7.1). After washing with PBS, samples were incubated with 1% osmium tetroxide in 0.1 M sodium cacodylate buffer before dehydration in a graduated ethanol series (30%, 50%, 70%, 90%, 95%, and 100%), followed by 100% acetone. Samples were critical-point dried with liquid carbon dioxide using a CPD 030 critical point dryer (BAL-TEC, Witten, Germany) and then sputter-coated with 20 nm gold particles using a SCD 005 (BAL-TEC). The cells were then examined under a Quanta 200 FEG field-emission scanning electron microscope (FEI, Eindhoven, The Netherlands). Three experiments were carried out in duplicate.

### 2.8. Bacterial Membrane Permeability Assay

*E. coli* strains CVCC1450 and *S. aureus* ATCC25923 were used in this assay. As previously described [19,20], bacteria were cultured to reach an OD_600_ value between 0.4 and 0.5. After centrifugation (10,000× *g*, 1 min), bacterial pellets were suspended in PBS to reach approximately 5 × 10^7^ CFU/mL. Suspensions were treated with various concentrations of *Z. morio* hemolymph for 1 h. Bacterial lysates were collected by centrifugation (5000× *g*, 10 min) and incubated with 10 μM of propidium iodide (PI) at 37 °C for 30 min in the dark. Samples were excited at 535 nm and OD_615_ were recorded. Bacterial lysates produced by ultrasonic treatment (300 W, 20 min) were used as positive control. For negative control, intact bacterial suspensions were incubated with PI; for blank control, only PI solution was used. Relative fluorescence intensity (RFI) was calculated as below:

(1) $$\begin{array}{c} RFI=\left(OD\;value\;of\;sample-OD\;value\;of\;blank\;control\right)\\ /\left(OD\;value\;of\;positive\;control-OD\;value\;of\;blank\;control\right). \end{array}$$

Three experiments were carried out in duplicate.

### 2.9. Hemolytic Activity Determination

As previously described [20], sheep erythrocytes were collected from fresh sheep blood by centrifugation (2000× *g*, 3 min, 4 °C). Erythrocytes were washed three times with PBS and adjusted to a stock concentration of 20%. For the hemolytic assay, 100 μL of erythrocyte stock was mixed with the same volume of *Z. morio* hemolymph (various concentrations starting from 0.2 mg/mL) and diluted with PBS to reach a total volume of 1 mL. Samples were incubated at 37 °C for 2 h. After centrifugation (2000× *g*, 3 min, 4 °C), the supernatants were removed and erythrocytes were hemolyzed by adding the same volume of 0.1% Triton X-100 (Sigma-Aldrich, St. Louis, MO, USA). The absorbance of the hemolyzed erythrocytes supernatants at 576 nm was measured. The level of hemolysis was considered as 0% for untreated erythrocytes samples and 100% for 0.1% Triton X-100 buffer. Three experiments were carried out in duplicate.

### 2.10. Adhesion Assay

MAC-T cells are a cell line derived from the bovine mammary epithelium, which were transferred with simian virus 40 (SV-40) T antigen [21] (a gift from Ying Yu, China Agricultural University). They were cultured in Dulbecco’s modified Eagle’s medium/Ham’s F-12 medium (1:1) supplemented with 5% fetal bovine serum, 100 U/mL of penicillin, and 0.1 mg/mL of streptomycin at 37 °C in an atmosphere of 5% CO_2_.

*E. coli* CVCC1450 or *S. simulans* No.11582 was cultured in LB broth overnight at 37 °C with constant shaking and subcultured 1:100 in fresh LB broth for an additional 3 h until reaching the mid-log phase (OD_600_ = 0.5).

For the adhesion assay, as previously described [22], MAC-T cells (3 × 10^5^ cells/well) were seeded into a 6-well cell culture plate (Corning, Inc., Corning City, NY, USA). Confluent cell monolayers were pretreated with 4 MIC of hemolymph (2 mg/mL for *E. coli* CVCC1450 and 4 mg/mL for *S. simulans* No.11582) for 1 h and then exposed to *E. coli* CVCC1450 (3 × 10^7^ CFU) or *S. simulans* No.11582 (3 × 10^7^ CFU). In a similar experimental setting, cell monolayers were treated with hemolymph and bacteria simultaneously. Then, 2 h after bacteria stimulation, cell monolayers were washed four times with PBS to remove nonadherent bacteria and treated with 0.05% trypsin for 10 min at 37 °C. Cells were harvested by centrifugation (4000× *g*, 10 min) and lysed with 100 μL of 0.2% Triton X-100 (Sigma-Aldrich, St. Louis, MO, USA) buffer. CFUs of *E. coli* or *S. simulans* were determined by LB agar plating. The adhesion rate of bacteria was determined by dividing the number of adhered bacteria treated with hemolymph to the number of adhered bacteria in the control group [23].

### 2.11. Internalization Assay

As previously described [22], MAC-T cells (3 × 10^5^ cells/well) were seeded into a 6-well cell culture. Confluent cell monolayers were pretreated with 4 MIC of hemolymph (4 mg/mL) for 1 h and then exposed to *S. simulans* No.11582 (3 × 10^7^ CFU). In a similar experimental setting, cell monolayers were treated with hemolymph and bacteria simultaneously. Internalizing events of *S. simulans* No.11582 were recorded after 2 h of co-incubation with *Z. morio* hemolymph followed by 2 h of culture with DMEM supplemented with gentamicin (100 μg/mL). After washing, digestion, and lysing, the supernatants were diluted and coated on a CAMH-A agar plate, and the CFU number was counted 18 h later.

### 2.12. Western Blot

MAC-T cells (3 × 10^5^ cells/well) were seeded into a 6-well cell culture plate and treated with *E. coli* CVCC1450 (3 × 10^7^ CFU) or *S. simulans* No.11582 (3 × 10^7^ CFU), in the presence or absence of *Z. morio* hemolymph (2 mg/mL for *E. coli* CVCC1450 and 4 mg/mL for *S. simulans* No.11582). Then, 8 h later, the whole cell lysis of each sample was collected and protein concentrations were determined with a bicinchoninic acid (BCA) protein assay kit (Pierce Chemical Co., Rockford, IL, USA), as previously described [22]. Primary antibodies including rabbit polyclonal anti-NLRP3 (19771-1-AP, 1:500 dilution; Protein Tech Group, Wuhan, Hubei, China), rabbit polyclonal anti-caspase-1 (ab179515, 1:500 dilution; Abcam, Cambridge, UK), goat polyclonal anti-NLRP6 (SC50639, 1:500 dilution; Santa Cruz Biotechnology, CA, USA), and mouse anti-GAPDH mAb (60004-1-Ig, 1:500 dilution; Protein Tech Group Wuhan, Hubei, China); and secondary antibodies including horseradish peroxidase-conjugated affinipure goat anti-mouse IgG (SA00001-1, 1:5000 dilution; Protein Tech Group Wuhan, Hubei, China) and goat anti-rabbit IgG (SA00001-2, 1:8000 dilution; Protein Tech Group Wuhan, Hubei, China) were used. Densitometric values of Western blot images were obtained from three independent experiments using Image J software (version 1.8.0, National Institutes of Health, Bethesda, MD, USA). Results are presented as the ratio of NLRP3, NLRP6, or caspase-1 intensity to GAPDH.

### 2.13. Cytotoxicity Determination

A cytotoxicity assay was performed based on the Enhanced Cell Counting Kit-8 (CCK-8) (Beyotime, Beijing, China). For detecting the pyroptosis induced by *E. coli* or *S. simulans*, MAC-T cells (5000 cells/well) were seeded into a 96-well cell culture plate and treated with *E. coli* or *S. simulans* (5 × 10^5^ CFU) in the presence or absence of *Z. morio* hemolymph (2 mg/mL for *E. coli* CVCC1450 and 4 mg/mL for *S. simulans* No.11582). Then, 18 or 24 h after stimulation, cells were washed with PBS and cultured in DMEM supplemented with 2% FBS and 10 μL of enhanced CCK-8 solution. After 2 h of culture, OD_450_ was measured. For detecting the cytotoxicity of *Z. morio* hemolymph, MAC-T cells (5000 cells/well) were seeded into a 96-well cell culture plate and treated with various concentrations of *Z. morio* hemolymph. Then, 2 h later, cells were washed with PBS and cultured in DMEM supplemented with 2% FBS and 10 μL of enhanced CCK-8 solution. After 2 h of culture, OD_450_ was measured.

### 2.14. Enzyme-Linked Immunosorbent Assay (ELISA)

MAC-T cell supernatants were collected 18 and 24 h after *E. coli* or *S. simulans* No.11582 infection, with or without *Z. morio* hemolymph (2 mg/mL for *E. coli* CVCC1450 and 4 mg/mL for *S. simulans* No.11582). Commercially available ELISA kits specific for bovine IL-1β (DG90995Q) and bovine IL-18 (DG91524Q; Beijing Dongge Biotechnology Co., Beijing, China) were used to detect the concentration of IL-1β and IL-18 in supernatants, respectively.

### 2.15. Caspase Substrates Cleavage Assay

MAC-T cells (3 × 10^5^ cells/well) were seeded into a 6-well cell culture plate and treated with *E. coli* CVCC1450 (3 × 10^7^ CFU) or *S. simulans* No.11582 (3 × 10^7^ CFU), in the presence or absence of *Z. morio* hemolymph (2 mg/mL for *E. coli* CVCC1450 and 4 mg/mL for *S. simulans* No.11582). After stimulation for 8 h, MAC-T cells were lysed with caspase assay buffer (50 mM HEPES pH 7.4, 100 mM NaCl, 0.1% CHAPS, 10 mM DTT, 1 mM EDTA, and 10% glycerol), and supernatants were collected after centrifugation (21,000 g, 2 min) and mixed with Ac-YVAD-pNA (YTB1001, BioLabBJ Company Ltd., Beijing, China) or Ac-LEVD-pNA (YTB1004, BioLabBJ Company Ltd., Beijing, China) (final concentration 200 μM) in a 96-well plate. The OD value was measured at 405 nm before or after 2 h of incubation at 37 °C. The level of increase in OD405 indicates the cleaving activity of caspases.

### 2.16. Statistical Analysis

Statistical analysis was performed by using GraphPad Prism7 software (version 7, GraphPad Software Inc., San Diego, CA, USA). For two-group comparisons with Gaussian distribution, a two-tailed unpaired *t*-test with Welch’s correction was applied when the variances of two groups were proved equal by the *F* test. For two-group comparisons with non-Gaussian distribution, a Mann–Whitney test was applied. For multigroup comparisons with Gaussian distribution, one-way ANOVA with Tukey-Kramer’s multiple-comparison test was used after the homogeneity of variance was confirmed by Bartlett’s test. For multigroup comparisons with non-Gaussian distribution, a Kruskal–Wallis test with Dunn’s test was used. *p* values of 0.05 or less were the threshold for statistical significance. *p*-values: * *p* < 0.05; ** *p* < 0.01; *** *p* < 0.001.

## 3. Results

### 3.1. Z. morio Hemolymph Shows Antibacterial Activity on Various Bacteria Strains

**Figure 1:**
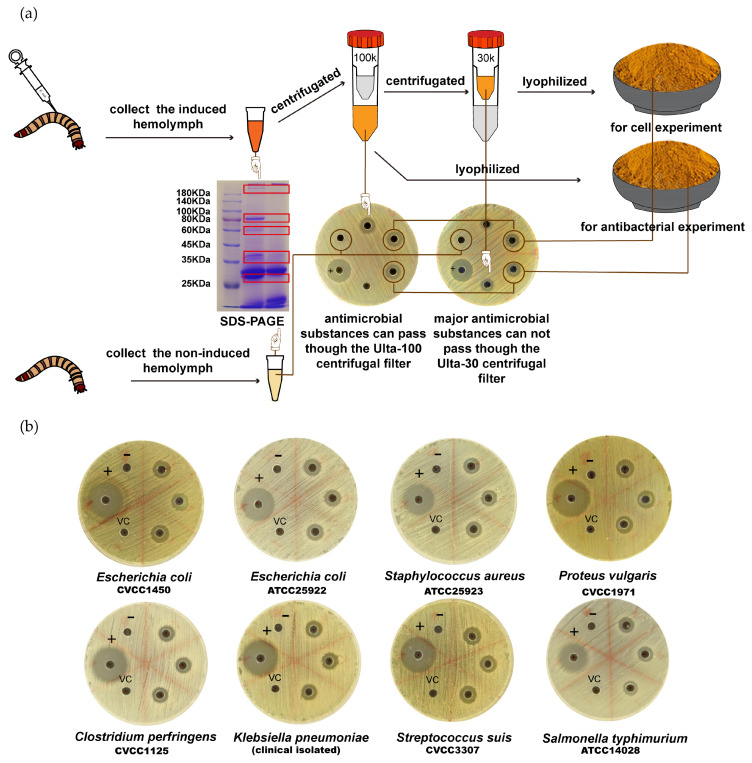
Preparation of antimicrobial hemolymph of *Z. morio* and the results of plate-based inhibition zone assay. (**a**) Workflow illustration of *Z. morio* hemolymph production. The red boxes represent bands that differ between the induced group and the noninduced group in the SDS-PAGE assay. (**b**) Susceptibility detection of bacteria to *Z. morio* hemolymph by disc agar diffusion method. The plus sign (+) indicates positive control (gentamicin 10 U/well); the minus sign indicates negative control. VC indicates vehicle control (saline solution 20 μL/well).

The process for *Z. morio* hemolymph production is illustrated in Figure 1a. Through SDS-PAGE analysis, there were different bands, most of which were between 25 and 100 KDa, in the hemolymph of the control group and induced group. Ultrafiltration experiments also confirmed that most of the antibacterial substances could pass through the Ulta-100 centrifugal filter, but not the Ulta-30 centrifugal filter (Figure 1a). This may indicate that the majority of antibacterial substances in hemolymph have large molecular weight or are polymers composed of small molecular substances.

From the bacterial susceptibility assay, we found that *Z. morio* hemolymph has a strong antibacterial effect on both Gram-positive and Gram-negative bacteria, especially for *E. coli*, *K. pneumoniae*, and *S. aureus*, which are the major causes of BM. By contrast, the bacteriostatic effect of *Z. morio* hemolymph on *p. vulgaris*, *C. perfringens*, and *S. typhimurium* is not obvious (Figure 1b).

To induce *Z. morio* hemolymph more efficiently, we tried different bacterial strains, in addition to *E. coli* CVCC1450 being shown to have the most capability; therefore, it was used for *Z. morio* hemolymph production in all the following experiments (Supplementary Table S1).

In order to quantify the sensitivity of different microbial strains to hemolymph, minimal inhibitory concentration (MIC) and minimum bactericidal concentration (MBC) assays were performed. MIC is defined as the lowest concentration of hemolymph that sufficiently inhibits the growth of the selected bacterial strain, while MBC is defined as the lowest concentration of *Z. morio* hemolymph that completely kills the selected bacterial strain. It shows that *Z. morio* hemolymph (induced by *E. coli* CVCC1450) is effective in inhibiting or killing all of the selected strains. The concentration of *Z. morio* hemolymph used in Gram-negative strains is lower for reaching the MIC or MBC (Table 1).

**Figure 2:**
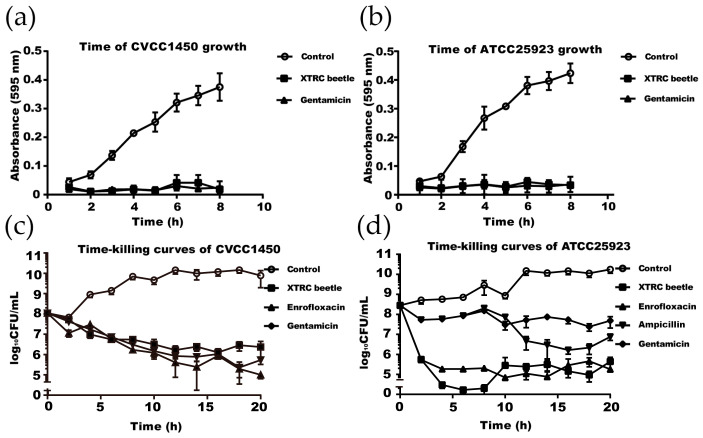
Timing course detection of *Z. morio* hemolymph in killing and inhibiting the growth of bacteria. (**a**–**d**) *E. coli* CVCC1450 (**a**) or *S. aureus* ATCC25923 (**b**) was incubated with 1 MIC of *Z. morio* hemolymph (0.5 mg/mL for *E. coli* and 1 mg/mL for *S. aureus*). 1 MIC of gentamicin (0.5 μg/mL) was used as a control drug in both assays. (**c**,**d**) *E. coli* CVCC1450 (10^8^ CFU/mL) and ATCC25923 (10^8^ CFU/mL) were incubated with 10 MIC of *Z. morio* hemolymph (5 mg/mL for *E. coli* CVCC1450 and 10 mg/mL for *S. aureus* ATCC25923). 10 MIC of gentamicin (5 μg/mL) and enrofloxacin (1.25 μg/mL) were used as control drugs in the *E. coli* group, and gentamicin (5 μg/mL), enrofloxacin (2.5 μg/mL), and ampicillin (20 μg/mL) were used as control drugs in the *S. aureus* group. XTRC beetle stands for *Z. morio* hemolymph in this figure. These experiments were carried out in triplicate and the results are expressed as the mean ± SD.

To further determine the antibacterial activity of *Z. morio* hemolymph, MIC and MBC assays were performed on *E. coli* CVCC1450 and *S. aureus* ATCC25923. Compared to the control, which has a significant increase in OD_595_ (indicator of bacterial growth), in a time-dependent manner, *Z. morio* hemolymph treatment completely inhibits growth of bacteria, similar to gentamicin treatment (the positive control). These results suggest that *Z. morio* hemolymph is effective in inhibiting the growth of *E. coli* and *S*. *aureus.* (Figure 2a,b).

In parallel, *E. coli* CVCC1450 and *S. aureus* ATCC25923 were incubated with 10 MIC of *Z. morio* hemolymph for a total of 20 h, and CFUs were determined at 2 h intervals (5 mg/mL for *E. coli* CVCC1450 and 10 mg/mL for *S. aureus* ATCC25923). The bactericidal activity of *Z. morio* hemolymph was observed as early as 4 h. Specifically, *Z. morio* hemolymph treatment significantly decreases the CFU number in the *S. aureus* group. These results indicate that the *Z. morio* hemolymph can effectively kill both Gram-positive and Gram-negative bacteria (Figure 2c,d, Supplementary Figure S1).

### 3.2. Validation of Z. morio Hemolymph’s Biostability

To determine the biostability, *Z. morio* hemolymph was treated under various extreme conditions including high temperature, repeated freeze–thaw, UV radiation, as well as lyophilization and dissolving. Bacterial susceptibility results show that even though affected, the overall antibiotic activity of *Z. morio* hemolymph remains active after any treatment (Supplementary Figure S2).

### 3.3. Hemolymph Induces Bacterial Membrane Permeabilization

**Figure 3:**
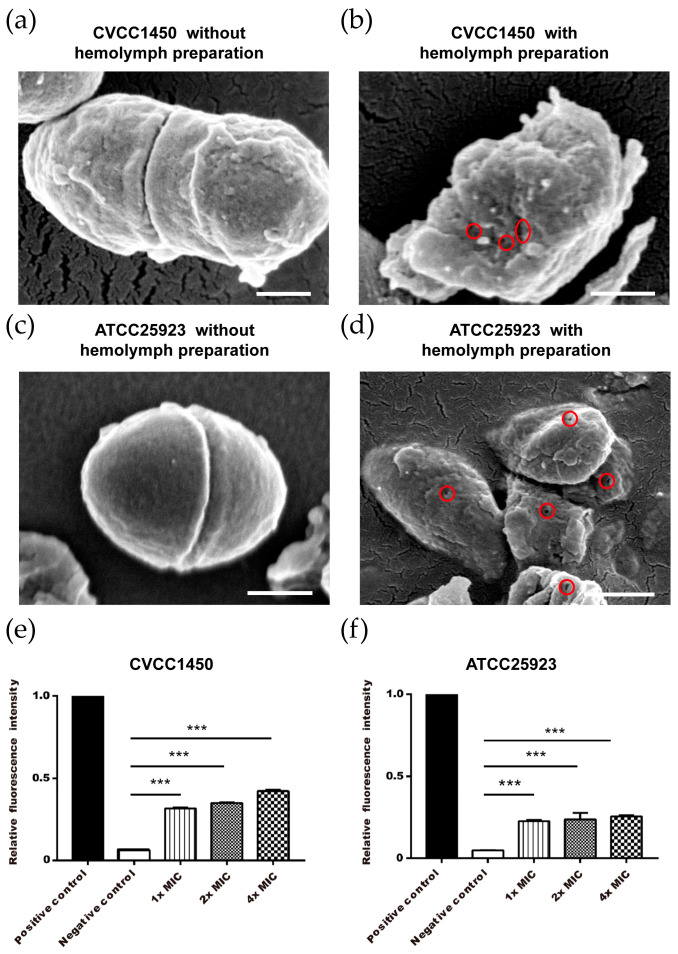
Bacterial membrane permeabilization induced by *Z. morio* hemolymph. (**a**,**b**) *E. coli* CVCC1450 (10^8^ CFU/mL) were incubated at 37 °C for 30 min with or without *Z. morio* hemolymph (1 mg/mL). Red circles indicate rupture of the cell wall integrity. Scale bars indicate 250 nm. (**c**,**d**) *S. aureus* ATCC25923 (10^8^ CFU/mL) were incubated at 37 °C for 30 min with or without *Z. morio* hemolymph (1 mg/mL). Red circles indicate rupture of the cell wall integrity. Scale bars indicate 250 nm. *E. coli* CVCC1450 (**e**) and *S. aureus* ATCC25923 (**f**) were treated with various concentrations of *Z. morio* hemolymph and incubated for 1 h at 37 °C followed by propidium iodide (PI) staining. Ultrasonic cracking lysed bacterial samples were used as a positive control and intact bacterial suspensions were used as a negative control. The blank control was similar to the negative control, except for no bacteria. Results were expressed as relative fluorescence intensity (RFI). RFI = (OD value of sample − OD value of blank control)/(OD value of positive control − OD value of blank control). Three experiments were carried out in duplicate and the results are expressed as the mean ± SD. *** *p* < 0.001.

Scanning electronic microscopy data show the formation of *E. coli* and *S. aureus* ultrastructure after hemolymph treatment, which suggests the potential membranolytic activity of *Z. morio* hemolymph (Figure 3a–d). To address this hypothesis, we assessed bacterial membrane permeabilization by PI staining. As shown, *Z. morio* hemolymph significantly increased the permeabilization of bacterial membrane as compared to the negative control (Figure 3e,f). Taken together, these results demonstrate the membranolytic activity of *Z. morio* hemolymph, which may contribute to bacterial death.

### 3.4. Hemolytic Activity and Cytotoxicity of Z. morio Hemolymph

**Figure 4:**
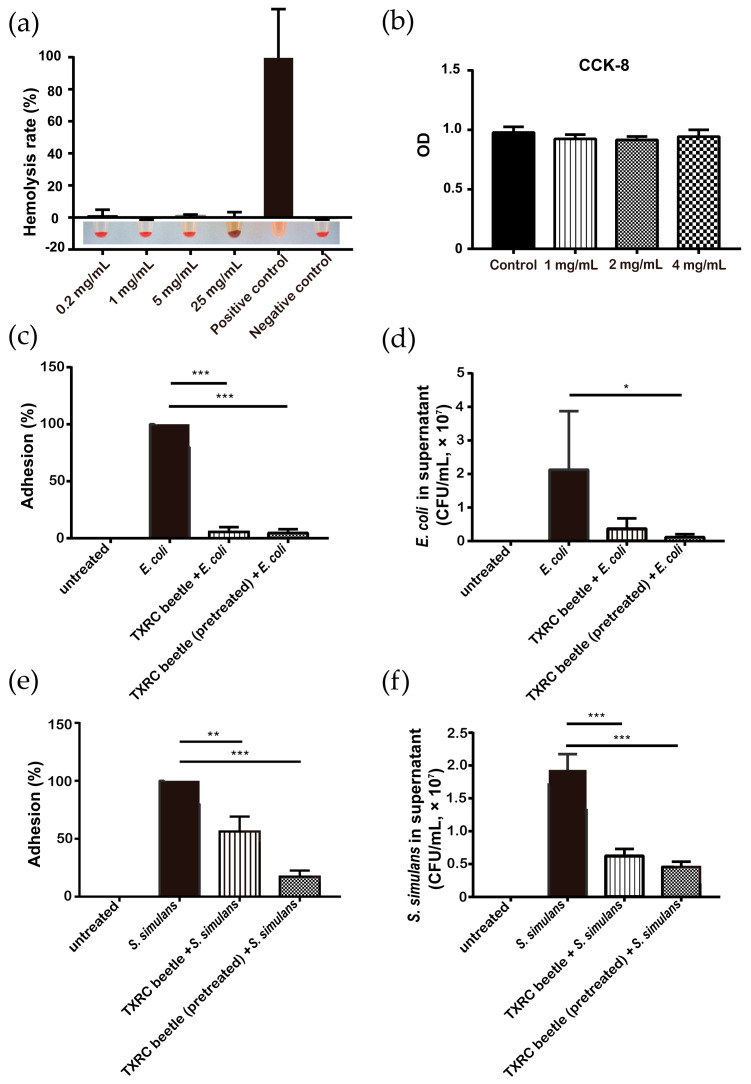
*Z. morio* hemolymph does not induce hemolysis and inhibits pathogens adhesion to MAC-T cells. (**a**) Erythrocytes were diluted to 2% in PBS buffer and incubated with various concentrations of *Z. morio* hemolymph for 2 h at 37 °C, and hemolysis rate was determined. (**b**) MAC-T cells (5000 cells/well) were incubated with various concentrations of *Z. morio* hemolymph, and cell pyroptosis was detected by the Cell Counting Kit-8 (CCK-8) assay 2 h later. (**c**,**e**) Adhesion assay of bacteria with MAC-T cells. (**c**) MAC-T cells (3 × 10^5^ cells/well) were either untreated or treated alone with *E. coli* (3 × 10^7^ CFU), treated with *E. coli* (3 × 10^7^ CFU) and *Z. morio* hemolymph (2 mg/mL) simultaneously (TXRC beetle + *E. coli*), and pretreated with *Z. morio* hemolymph (2 mg/mL) for 1 h and then followed by *E. coli* (3 × 10^7^ CFU). The percentage of adhesions of each group was accessed 2 h later. (**e**) Similar to (**c**), except that *S. simulans* was used instead of *E. coli* and the concentration of *Z. morio* hemolymph was 4 mg/mL. (**d**,**f**) CFU detection in co-cultured supernatants. (**d**) Similar to (**c**), except the CFU in supernatant was detected. (**f**) Similar to (**e**), except the CFU in supernatant was detected. For all panels, XTRC beetle stands for *Z. morio* hemolymph in this figure. These experiments were carried out in triplicate and the results are expressed as the mean ± SD. * *p* < 0.05, ** *p* < 0.01, *** *p* < 0.001.

To investigate the effect of *Z. morio* hemolymph on the mammalian cell membrane, a hemolytic assay was performed by incubating hemolymph with sheep erythrocytes. As shown, there is no observable hemoglobin release after hemolymph treatment, which excludes the possibility that *Z. morio* hemolymph lyses the erythrocyte membrane (Figure 4a).

Similarly, there is no significant difference of cell counts in MAC-T cells treated with hemolymph or left untreated. The data suggest that hemolymph may be well-tolerated in the cattle mammary gland without evident toxicity (Figure 4b).

### 3.5. Z. morio Hemolymph Reduced the Adhering Activity of E. coli and S. simulans

The ability of adhering to the cell surface is a prerequisite for bacterial infection and BM pathogenesis. In order to understand whether *Z. morio* hemolymph treatment inhibits the adhering activity of pathogenic bacteria, in this assay, we used *E. coli* CVCC1450, a pathogenic strain widely used in the BM cell model, and *S. simulans* No.11582, the most severe one of six isolated *Staphylococcus* strains (Supplementary Figure S3) to investigate.

As shown, the number of adhesive *E. coli* and *S. simulans* was 4.57 × 10^4^ ± 3.14 × 10^4^ CFU and 1.93 × 10^7^ ± 2.41 × 10^6^ CFU 2 h after infection, respectively. Specifically, treatment with hemolymph significantly reduced the percentage of adhering *E. coli* cells (*p* < 0.001) and was further reduced by hemolymph pretreatment (5.54% vs. 4.72%) (Figure 4c). A similar pattern was shown in *S. simulans* infection and the difference between hemolymph treatment and pretreatment was also obvious (56.4% vs. 17.3%). (Figure 4e). In addition, the number of *E. coli* or *S. simulans* recovered from supernatant fractions in the adhesion assay was determined, and a significant difference was also observed between treated and untreated groups (Figure 4d–f).

The effect of *Z. morio* hemolymph on the internalization rate of *S. simulans* was also determined. It shows that pretreatment or simultaneous treatment of hemolymph reduced the internalization rate of *S. simulans* to MAC-T cells by 39.3% and 37.3%, respectively (Supplementary Figure S4).

### 3.6. Z. morio Hemolymph Attenuates E. coli or S. simulans-Induced IL-1β and IL-18 Production and Improves Cell Viability

**Figure 5:**
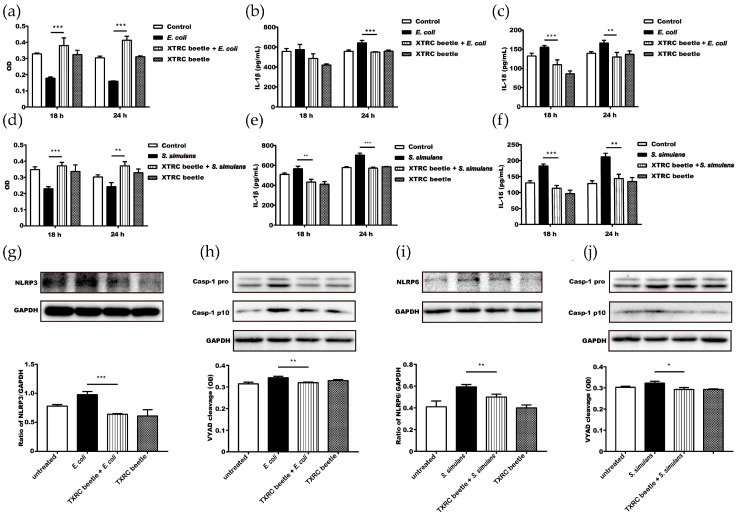
*Z. morio* hemolymph inhibits bacteria-induced NLRP3 and inflammasome activation. (**a**) MAC-T cells (5000 cells/well) were either untreated; treated alone with *E. coli* (5 × 10^5^ CFU)*;* treated with *Z. morio* hemolymph (2 mg/mL) and *E. coli* (5 × 10^5^ CFU); treated alone with *Z. morio* hemolymph (2 mg/mL). 18 or 24 h after stimulation, cells were washed with PBS and cultured in DMEM supplemented with 2% FBS and 10 μL of enhanced CCK-8 solution. After 2 h of culture, OD_450_ was measured. (**b**) MAC-T cells (3 × 10^5^ cells/well) were either untreated; treated alone with *E. coli* (3 × 10^7^ CFU)*;* treated with *Z. morio* hemolymph (2 mg/mL) and *E. coli* (3 × 10^7^ CFU); treated alone with *Z. morio* hemolymph (2 mg/mL). 18 or 24 h after stimulation, supernatants were collected for detecting the concentration of IL-1β. (**c**) Similar to (**b**), except the supernatants were collected for detecting the concentration of IL-18. (**d**) MAC-T cells (5000 cells/well) were either untreated; treated alone with *S. simulans* (5 × 10^5^ CFU)*;* treated with *Z. morio* hemolymph (4 mg/mL) and *S. simulans* (5 × 10^5^ CFU); treated alone with *Z. morio* hemolymph (4 mg/mL). 18 or 24 h after stimulation, cells were washed with PBS and cultured in DMEM supplemented with 2% FBS and 10 μL of enhanced CCK-8 solution. After 2 h of culture, OD_450_ was measured. (**e**) MAC-T cells (3 × 10^5^ cells/well) were either untreated; treated alone with *S. simulans* (3 × 10^7^ CFU)*;* treated with *Z. morio* hemolymph (4 mg/mL) and *S. simulans* (3 × 10^7^ CFU); treated alone with *Z. morio* hemolymph (4 mg/mL). 18 or 24 h after stimulation, supernatants were collected for detecting the concentration of IL-1β. (**f**) Similar to (**e**), except the supernatants were collected for detecting the concentration of IL-18. (**g**) Similar to (**b**), except that 8 h after challenging, the whole cell lysis was used for detecting the NLRP3. (**h**) Similar to (**b**), except 8 h after challenging, the whole cell lysis was used for detecting the pro-caspase-1 and caspase-1 p10. (**i**) Similar to (**e**), except that 8 h after challenging, the whole cell lysis was used for detecting the NLRP6. (**j**) Similar to (**e**), except that 8 h after challenging, the whole cell lysis was used for detecting pro-caspase-1 and caspase-1 p10. XTRC beetle stands for *Z. morio* hemolymph in this figure. Data are presented as the mean ± SD of three independent experiments. * *p* < 0.05, ** *p* < 0.01, *** *p* < 0.001.

Proinflammatory cytokines like IL-1β and IL-18 are effector molecules produced during the process of pyroptosis. The level of those cytokines indicates the severity of pyroptosis and inflammation. It is shown that cells treated with *Z. morio* hemolymph produced significantly less IL-1β and IL-18 as compared to the infected-only group 18 and 24 h post-infection (Figure 5b,c,e,f). CCK-8 tests showed that hemolymph treatment significantly increased the viability of cells 18 and 24 h post-infection (Figure 5a,d).

### 3.7. Z. morio Hemolymph Attenuates E. coli-Induced NLRP3 and S. simulans-Induced NLRP6 Expression

NLRP3 and NLRP6 inflammasomes are important players in bacterial-induced inflammation, pyroptosis, and IL-1β/IL-18 production. To investigate the role of *Z. morio* hemolymph in regulating NLRP3 and NLRP6 inflammasome activation, we first compared the expression level of NLRP3 and NLRP6 under different conditions. As shown, the expression level of NLRP3 was significantly lower in the hemolymph-treated group (2 mg/mL for *E. coli* and 4 mg/mL for *S. simulans*) than the control group after *E. coli* infection (Figure 5g). Similarly, hemolymph treatment significantly reduced the expression of NLRP6 *S. simulans* infection (Figure 5i).

### 3.8. Z. morio Hemolymph Attenuates E. coli or S. simulans-Induced Caspase-1 or Caspase-4 Cleavage

The cleavage and activation of caspase-1 followed by NLRP3 or NLRP6 recruitment is an essential step in pyroptosis and pro-inflammatory cytokine production; therefore, we also tested the effect of *Z. morio* hemolymph (2 mg/mL for *E. coli* and 4 mg/mL for *S. simulans*) on caspase-1 cleavage. As shown, the level of caspase-1 cleavage is significantly lower in the *Z. morio* hemolymph treatment group than the control (Figure 5h,j).

As a homolog of human caspase-4 and mouse caspase-11, Bovine caspase-4 also plays important role in maturation of IL-1β and IL-18 [24]. Our data show that the level of cleaved caspase-4 was significantly lower in the *Z. morio* hemolymph-treated group than the control group after *E. coli* or *S. simulans* infection (Supplementary Figure S5).

## 4. Discussion

It is well-known that antibiotic peptides are widely expressed in most insect species [25]. *Harmonia axyridis*, an Asian lady beetle, was reported to express an abundant level of various kinds of antimicrobial peptides [26]. In our study, as compared to the control, *E. coli*-induced hemolymph extracts showed multiple additional bands in SDS-PAGE analysis. Therefore, it is reasonable to assume the high diversity of antimicrobial molecules in *E. coli*-induced *Z*. *morio* hemolymph. In addition, as reported previously, there were three different antimicrobial compounds identified in *Z. atratus* (another species closely related to *Z. morio*)—coleoptericin, defensins B, and defensins C [18]—so it is reasonable to assume that similar compounds existed *Z. morio* as well. Ultrafiltration separation and SDS-PAGE analysis suggested that the approximate molecular weight of effective antimicrobial compounds is between 30 and 100 kDa. With improved techniques like 2-D electrophoresis or high-performance liquid chromatography (HPLC), more compounds with antibacterial activity would be identified. More importantly, *E. coli*-induced *Z. morio* hemolymph showed a broad antibacterial profile against *E. coli*, *S. aureus*, *K. pneumoniae*, as well as other commonly seen pathogens in BM, which suggests its potential capability as a future drug candidate.

Our data show that *Z. morio* hemolymph is effective in killing *E. coli* or *S. aureus*, in addition to gentamicin and enrofloxacin. SEM and membrane permeability results revealed the bactericidal activity of hemolymph, which is consistent with the role of other arthropod-derived antimicrobial peptides, as previously reported [20]. In general, *Z. morio* hemolymph is worthwhile to be developed in both preventing and curing BM.

From the perspective of drug development, biostability is undoubtedly an important factor in addition to effectivity. Gramicidin A, a proved antimicrobial peptide drug clinically, can only be used topically due to its hemolytic activity [27]. As a common side-effect, hemolysis largely compromised the application of most antimicrobial peptides [19,28]. Although considered an in vitro environment, it is believed that the inactivity of hemolysis is also a pre-requirement for all drugs targeting lactiferous ducts. Our data demonstrate that *Z. morio* hemolymph has no obvious hemolytic activity or cytotoxicity in the range of effective concentrations. Besides, the biostability of *Z. morio* hemolymph under various extreme conditions was also assured. Overall, these results strongly suggest that *Z. morio* hemolymph would be a promising candidate for antimicrobial drugs.

As described above, *E. coli* and *S. aureus* are two of the major pathogens inducing BM, and *E. coli* CVCC1450 is the most commonly used strain in BM studies [29,30]. *S. simulans*, a non-*aureus staphylococci* species, is also responsible for subclinical BM [31]. In addition, *S. simulans* was reported to induce osteoarticular and dermatic infection in human [32]. Our data show that *S. simulans* has the most cytotoxicity in various Staphylococcus species tested. Therefore, in order to investigate the protective role of *Z. morio* hemolymph in mammary epithelial cells under infection, we used both *E. coli* CVCC1450 and *S. simulans* (Supplementary Figure S3).

Regarding the molecular mechanism of *Z. morio* hemolymph’s protective effect during bacterial infection, we found that *Z. morio* hemolymph efficiently downregulates the expression of inflammasome-related genes including NLRP3 and NLRP6, which are reported as important players in bacterial sensing and inflammasome activation [10,11]. Besides, *Z. morio* hemolymph treatment also attenuates cleavage of caspase-1 and caspase-4, and the secretion of mature IL-1β and IL-18 was also inhibited. These data illustrated the regulating mechanisms of *Z. morio* hemolymph. Thus, future work on compound purification and functional determination will provide a deeper insight on molecular interactions and signal transduction. Overall, our study highlights the potential role of *E. coli*-induced *Z. morio* hemolymph in BM protection, which promotes the investigation of antimicrobial activity from natural extracts and the development of new treatments in response to bacterial infection-induced BM.

## Figures and Tables

**Table 1:** Minimum inhibitory concentration (MIC) and minimum bactericidal concentration (MBC) values of *Z. morio* antimicrobial hemolymph.

Microbial Strains		MIC of Hemolymph (mg/mL)	MBC of Hemolymph (mg/mL)
Gram-Positive	*S. aureus* ATCC25923	1	1
	*S. suis* CVCC3307	1	2
	*S. simulans* No.11582	1	1
	*C. perfringens* CVCC1125	2	4
Gram-Negative	*E. coli* ATCC25922	0.25	0.25
	*E. coli* CVCC1450	0.5	1
	*P. vulgaris* CVCC1971	0.5	2
	*K. pneumoniae* (clinical isolated)	0.5	1

## Supplementary Materials

Supplementary materials can be found at https://www.mdpi.com/2076-2607/8/10/1488/s1, Figure S1: Timing course detection of *Z. morio* hemolymph in killing bacteria, Figure S2: Biostability determination of *Z. morio* hemolymph, Figure S3: *S. simulans* No.11582 has the strongest cytotoxicity among all tested Staphylococcus species, Figure S4: *Z. morio* hemolymph inhibits internalization of *S. simulans* No.11582, Figure S5: Cleavage of caspase-4 was decreased by *Z. morio* hemolymph treatment, Table S1: Antibacterial hemolymphs from *Z. morio* induced by different stimulations.
