# Supplementary material for: Antimicrobial Effect of Zophobas morio Hemolymph against Bovine Mastitis Pathogens
Source: Microorganisms. 2020 Sep 28;8(10):1488. doi: 10.3390/microorganisms8101488 (PMC7601528; doi:10.3390/microorganisms8101488)
Supplement: Supplementary file 1 [file microorganisms-08-01488-s001.pdf]

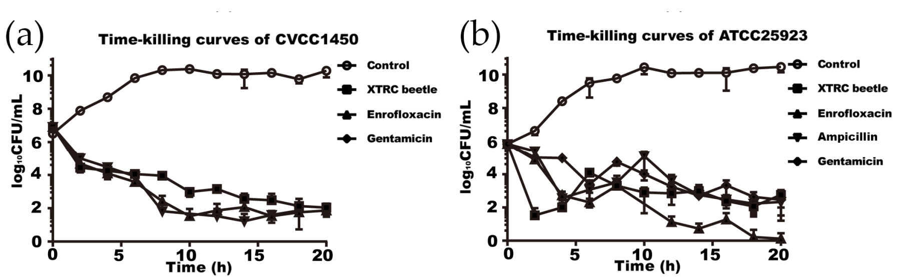

Figure S1. Timing course detection of *Z. morio* hemolymph in killing bacteria. (**a**-**d**). *E. coli* CVCC1450 (10^6 CFU/mL) and ATCC25923 (10^8 CFU/mL) were incubated with 10 MIC of *Z. morio* hemolymph (5 mg/mL for *E. coli* CVCC1450 and 10 mg/mL for *S. aureus* ATCC25923). 10 MIC of gentamicin (5 μg/mL) and enrofloxacin (1.25 μg/mL) were used as control drugs in the *E. coli* group, and gentamicin (5 μg/mL), enrofloxacin (2.5 μg/mL), and ampicillin (20 μg/mL) were used as control drugs in the *S. aureus* group. XTRC beetle stands for *Z. morio* hemolymph in this figure. These experiments were carried out in triplicate and the results are expressed as the mean ± SD.

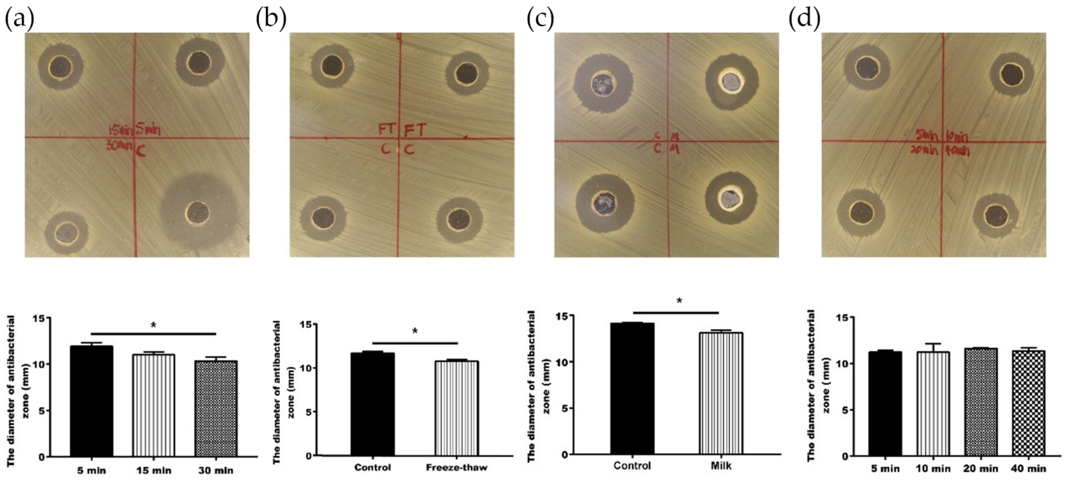

Figure S2. Biostability determination of *Z. morio* hemolymph. **(a)** *Z. morio* hemolymph was boiled for 5, 15, or 30 min, and the antimicrobial activity against *E. coli* CVCC1450 was determined by susceptibility assay. **(b)** Similar to (**a**) except *Z. morio* hemolymph was treated by 6 cycles of freezing-thaw repeats. **(c)** Similar to (**a**) except *Z. morio* hemolymph was dissolved in milk. **(d)** Similar to (**a**) except *Z. morio* hemolymph was treated under UV radiation for different times. For all panels, “C” is for control group, “FT” is for freezing-thaw group, “M” is for milk-dissolved *Z. morio* hemolymph treating group. Data are presented as the mean ± SD of three independent experiments.

(a)

	Description	Max Score	Total Score	Query Cover	E value	Per. Ident	Accession
☑	Staphylococcus simulans strain CD208 16S ribosomal RNA gene, partial sequence	1380	1380	96%	0.0	100.00%	MG798677.1

(b)

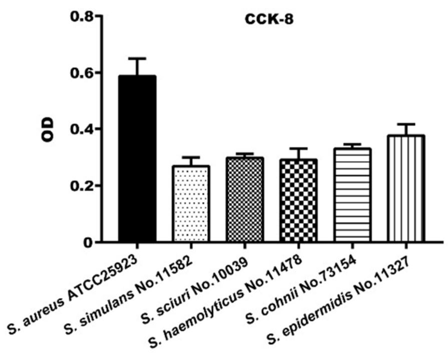

Figure S3. *S. simulans* No.11582 has the strongest cytotoxicity among all tested Staphylococcus species. **(a)** BLAST result based on 16S ribosomal RNA sequence comparison. **(b)** Different virulence of Staphylococcus spp. on MAC-T cells determined by CCK-8 assay. Data are presented as the mean ± SD of three independent experiments.

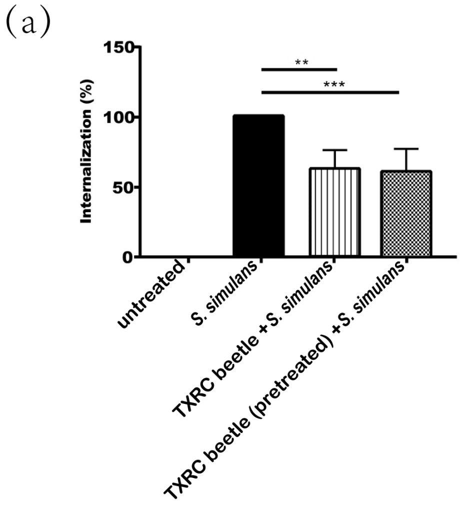

Figure S4. *Z.morio* hemolymph inhibits internalization of *S.simulans* No. 11582. MAC-T cells (3 × 10^5 cells/well) were either untreated or treated alone with *S.simulans* (3 × 10^7 CFU), treated with *S.simulans* (3 × 10^7 CFU) and *Z.morio* hemolymph (4 mg/mL) simultaneously (TXRC beetle + *S.simulans*), and pretreated with *Z.morio* hemolymph (4 mg/mL) for 1 h followed by *S.simulans* (3 × 10^7 CFU). 2 hours later, cells were washed and lysed in 0.001% triton, cells lysates were diluted, and internalized bacteria were quantified by CFU counting 18 h later. Data are presented as the mean ± SD of three independent experiments. ***$P < 0.001$.

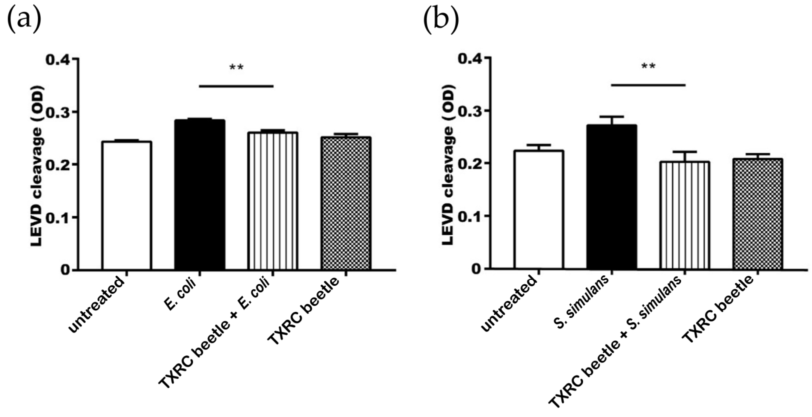

Figure S5. Cleavage of caspase-4 was decreased by *Z. morio* hemolymph treatment. **(a)** MAC-T cells (3 × 10^5 cells/well) were either untreated or treated alone with *E. coli* (3 × 10^7 CFU); treated with *Z. morio* hemolymph (2 mg/mL) and *E. coli* (3 × 10^7 CFU); treated alone with *Z. morio* hemolymph (2 mg/mL). 8 hours later, caspase-4 cleavage was detected. **(b)** Similar to Figure S4a except that *S. simulans* was used instead of *E. coli* and the concentration of *Z.morio* hemolymph was 4 mg/mL. Data are presented as the mean ± SD of three independent experiments. **$P < 0.01$.

Table S1. Antibacterial hemolymphs from *Z. morio* induced by different stimulations. Numbers in this table stand for MIC values of antimicrobial hemolymph (mg/mL) from *Z. morio* induced by different bacteria.

Stimulations	CVCC 1450	ATCC 25922	*K. pneumoniae*	CVCC 1971	ATCC 25923	CVCC 3307	*B. subtilis*	*B. licheniformis*	*L. rhamnosus* GR-1
CVCC1450	0.5	0.25	0.5	0.5	1	1	1	>8	0.5
ATCC25922	1	0.25	1	1	1	2	1	>8	2
K. pneumoniae	0.5	0.5	>8	>8	1	>8	2	>8	1
CVCC1971	0.5	1	>8	>8	1	>8	1	>8	1
ATCC25923	0.5	0.25	4	>8	1	>8	1	>8	0.5
CVCC3307	2	1	>8	>8	1	>8	2	>8	4
B. subtilis	2	1	>8	>8	1	>8	>8	>8	1
B. licheniformis	2	0.5	>8	>8	1	>8	>8	>8	4
L. rhamnosus GR-1	>8	>8	>8	>8	>8	>8	>8	>8	>8
MHA medium									
